# Identification of prognostic biomarkers for endometrioid endometrial carcinoma based on the miRNA and mRNA co‐expression network regulated by estradiol

**DOI:** 10.1016/j.clinsp.2025.100672

**Published:** 2025-05-03

**Authors:** Qiu Xie, Junting Huang, Yuan Xie, Jin Hu, Li Jin

**Affiliations:** a Department of Medical Research Center, State Key Laboratory for Complex Severe and Rare Diseases, Peking Union Medical College Hospital, Chinese Academy of Medical Sciences and Peking Union Medical College, PR China; b National Clinical Research Center for Obstetric and Gynecologic Diseases, Department of Obstetrics and Gynecology, Peking Union Medical College Hospital, Chinese Academy of Medical Sciences and Peking Union Medical College, PR China

**Keywords:** Endometrioid endometrial carcinoma, MicroRNA, Molecular Biomarker, Prognostic Model

## Abstract

**Background:**

Endometrioid Endometrial Carcinoma (EEC), an estradiol-related disease, remains a serious health threat to women because of its high incidence and trend of rejuvenation. Accumulating evidence has highlighted that microRNAs (miRNAs) and messenger RNAs (mRNAs) play important roles in various biological processes involved in the pathogenesis of EEC. This study aimed to identify the potential prognostic biomarkers associated with EEC regulated by estradiol.

**Methods:**

RNA expression profiles of EEC were obtained from The Cancer Genome Atlas database (*n* = 408) and the original sequencing, which was performed on endometrial cancer Ishikawa cells treated with 250 nM estradiol (*n* = 3), 50 nM estradiol (*n* = 3) or control (*n* = 3). The TargetScan database was used to predict the target genes of prognosis-related differentially expressed miRNAs (DEMs). Subsequently, functional enrichment analysis and topological analysis were performed on the overlaps of target genes and differentially expressed mRNAs (DEGs). Kaplan-Meier analysis was used to predict prognosis‐related target genes to identify prognostic biomarkers and cell population landscapes, and gene expression analysis was performed to locate prognosis-related DEGs based on single-cell transcriptomic sequencing data from the NCBI Sequence Read Archive database.

**Results:**

Four estradiol-related DEGs were associated with prognosis, and 235 overlapping target DEGs were screened and incorporated into the functional enrichment analysis and protein-protein interaction network visualization studies. Additionally, SACS and GPR157 were identified as potential biomarkers for EEC prognosis through survival analyses. Furthermore, single-cell transcriptome data were analyzed to show changes in gene expression levels in specific cell types.

**Conclusions:**

This study demonstrates that miR-142–5p–SACS and miR-30a-5p–GPR157, which are regulated by estradiol, may hold promise as diagnostic and prognostic biomarkers and novel therapeutic targets for EEC.

## Highlights

• This study aimed to identify prognostic biomarkers for Endometrioid Endometrial Carcinoma (EEC) regulated by estradiol.
• miR-142–5p and miR-30a-5p exhibited high sensitivity to estradiol stimulation and a strong association with EEC prognosis.
• SACS and GPR157 were identified as potential biomarkers for EEC prognosis.
• miR-142–5p–SACS and miR-30a-5p–GPR157 are novel therapeutic targets for EEC.

## Introduction

Endometrial Cancer (EC), the most common malignant tumor of the female reproductive tract in developed countries, has a rising incidence and mortality rate, posing a significant threat to public health^1^^,^^2^ Despite improvements in the treatment and diagnosis of EC, advanced stages of the disease are still difficult to manage, with a 5-year survival rate of 10%–29%.^3^ EC encompasses various histological subtypes that differ markedly in pathogenesis, clinical presentation, and prognosis.^1^

Endometrioid Endometrial Carcinoma (EEC), the most common histological type of EC, constitutes 80% of newly diagnosed cases and is widely considered an estrogen-dependent tumor.^4^ The pathogenesis of EEC has been linked to mutations in several genes, including TP53, PTEN, AKT, mTOR, KRAS, and CTNNB1.^5^, ^6^, ^7^, ^8^ However, these alterations were not found uniformly in all cases of EEC. Therefore, the discovery of new uniform biomarkers would help to improve diagnosis and treatment and reduce the risk of death in patients with EEC.

MicroRNAs (miRNAs) are small noncoding RNAs that can impede the translation and stability of messenger RNAs (mRNA) usually by base-pairing to the 3′-untranslated regions of mRNA.^9^ Due to their physical and chemical properties, such as remarkable stability in body fluids and formalin-fixed paraffin-embedded tissues, miRNAs represent a promising class of potential diagnostic and prognostic markers.^10^

High-throughput microarray technologies and molecular dynamics analyses provide new insights into gene expression changes during tumorigenesis.^11^, ^12^, ^13^ Integrating molecular biology with computational tools allows for the precise identification of cancer targets, facilitating the discovery of biomarkers for cancer diagnosis, prognosis, and targeted therapy.^14^, ^15^, ^16^

Although some studies have suggested the involvement of miRNAs in the pathogenesis of EC, only a few involved large groups of patients and addressed issues concerning the influence of estrogen on miRNA expression in EEC. Similarly, the prognostic accuracy of miRNA signatures in the plasma of patients with EEC has not yet been systematically studied. Therefore, the authors aimed to elucidate the regulatory mechanisms of miRNAs in the prognosis of EEC to identify novel biomarkers for early diagnosis and personalized treatment.

## Methods

### High-throughput sequencing and ethical approval

RNA sequencing was performed on endometrial cancer Ishikawa cells treated with 250 nM estradiol (*n* = 3), 50 nM estradiol (*n* = 3) or control (*n* = 3). Total RNA was isolated using a TRIzol reagent (Invitrogen, Carlsbad, CA, USA), according to the manufacturer’s recommendations. RNA-seq experiments were conducted in triplicate. Individual runs showed a high degree of correlation with the promoters. Sequencing reads were trimmed using Trim Galore (version 0.6.0), a Perl wrapper based on Cutadapt and FastQC. Reads with a quality > 20 were aligned to the reference genome (mm 10) using TopHat2 (version 2.1.1). Gene expression levels were calculated using the Cufflinks software (version 2.2.1). The relative abundance of the transcripts was determined as fragments per kilobase of exons per million mapped reads. All raw and processed sequencing data generated in this study were submitted to the NCBI Gene Expression Omnibus (https://www.ncbi.nlm.nih.gov/geo/) under the accession number GSE274274. The study and all experimental procedures were approved by the hospital’s Ethics Committee, according to the Council for International Organizations of Medical Sciences (JS-2241).

### External data collection

The mRNA-seq, miRNA-seq, and relevant clinical data of patients with EEC were downloaded from The Cancer Genome Atlas (TCGA) (www.cancergenome.nih.gov). The mRNA-seq and miRNA-seq datasets contained 408 tumor samples and 20 adjacent normal tissue samples, and 408 tumor samples and 19 adjacent normal tissue samples, respectively. Additionally, miRNA expression profiles for human EEC were obtained from the Gene Expression Omnibus (GEO) dataset GSE35794 for external validation, including 11 EEC samples and 3 normal controls. Single-cell transcriptome analysis of EEC was downloaded from the NCBI Sequence Read Archive database (https://www.ncbi.nlm.nih.gov/) under accession number SRP349751, which contains endothelial tissue samples from patients with EEC (*n* = 5) and healthy controls (*n* = 5). The downloaded data were statistically analyzed using R‐studio software 4.3.1(http://www.R-project.org).

### Differential expression analysis

Bioconductor packages were used to identify the dysregulated genes in EEC and adjacent normal tissues. Differential expression of mRNAs (DEGs) and miRNAs (DEMs) were identified using the “limma” R package (version 3.40.6) with a False Discovery Rate (FDR) adjusted *p* < 0.051 and |log fold change (FC)| > 1.0 as the screening criteria, respectively. In addition, the R package of ggplot2 was used to generate a volcano map.

### Functional enrichment analysis with GO annotations and KEGG signaling pathways

The R package “Cluster Profiler” (version 3.14.3) was used to perform Gene Ontology (GO) and Kyoto Encyclopedia of Genes and Genomes (KEGG) pathway enrichment analyses of the identified DEGs. The GO analysis included the Biological Process (BP), Cellular Component (CC), and Molecular Function (MF) terms. In addition, the Benjamin-Hochberg method was used to obtain the FDR. Gene sets were considered significantly enriched under a threshold of *p* < 0.05 and an FDR < 0.1.

### Survival analysis and definition of the miRNA‐related prognostic signature

To identify prognosis-related miRNAs and mRNAs, the authors adjusted and standardized the FDR values of DEMs and DEGs. The prognosis model (univariate and multivariate Cox regression analysis) was analyzed using the “Survival” and “Survminer” R packages. Nomograms were used to predict cancer prognosis. The “ggplot2” and “RMS” R packages were used to visualize the analysis results of the prognosis model. Statistical significance was set at *p* < 0.05.

### Protein-protein interaction (PPI) and miRNA-mRNA network construction

PPI network information with a combined score of 0.4 was obtained from the Search Tool for the Retrieval of Interacting Genes database (http://string-db.org) based on uploaded DEGs. The degree score of each gene was obtained using the Network Analyzer tool of Network Analyzer in Cytoscape software 3.10.2 (https://cytoscape.org). Then, genes with a degree ≥ 10 were selected to be present in the PPI network. The miRNA-mRNA network was visualized using Cytoscape. The target genes of the miRNAs were predicted using three online analysis tools, including TargetScan.

### Statistical analysis

In this study, R version 4.3.1 was used for statistical analysis. The statistical tests used to evaluate each set of data are mentioned in the figure legends and include a two-tailed Student’s *t*-test, one-way analysis of variance, Pearson correlation analysis, univariate Cox regression, multivariate Cox regression, and Least Absolute Shrinkage and Selection Operator (LASSO) Cox regression. Most quantitative data are presented as mean ± standard error of the mean (SEM); *p* < 0.05 was considered statistically significant.

## Results

### Identification of differentially expressed miRNAs and potential prognosis-related miRNAs in EEC

**Fig. 1:**
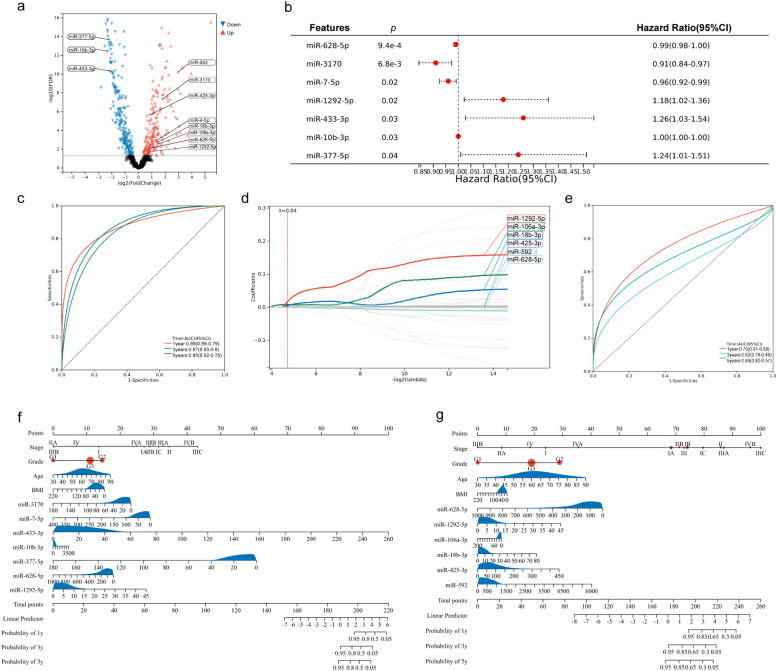
Identification of differentially expressed miRNAs and prognosis-related miRNAs of endometrioid endometrial carcinoma (EEC). (a) Volcano plot of differentially expressed miRNAs (DEMs) in EEC. (b) The multivariate Cox regression analysis showed seven potential prognosis-related DEMs (miR-628–5p, miR-3170, miR-7–5p, miR-1292–5p, miR-433–3p, miR-10b-3p, and miR-377–5p) in EEC. (c)The Receiver Operating Characteristic (ROC) curve of the multivariate Cox regression model with seven miRNAs in EEC. (d) Coefficient profile plot was generated at the selected log λ value using a 10-fold cross-validation, six miRNAs (miR-628–5p, miR-1292–5p, miR-106a-3p, miR-18b-3p, miR-425–3p, miR-592) with the best coefficients were selected. (e) The ROC curve of the LASSO regression model with six miRNAs in EEC. (f‒g) The nomogram shows the prognostic model of the multivariate Cox regression analysis and the lasso Cox regression analysis.

A total of 427 miRNA samples were obtained from the TCGA database, including 408 EEC tumor tissues and 19 adjacent normal tissues. Analysis of the miRNA expression profiles in EEC revealed that 236 miRNAs were upregulated, and 240 miRNAs were downregulated (|log FC| > 1.0, *p* < 0.05) after FDR adjustment (Fig. 1a).

To investigate the impact of these DEMs on the prognosis of EEC, an effective model was established for predicting the prognostic status using univariate and multivariate Cox proportional hazards regression analyses. In the univariate Cox proportional hazards regression analysis, 54 miRNAs were selected as potential prognostic biomarkers. The multivariate Cox proportional hazards regression analysis showed that miR-628–5p, miR-3170, miR-7–5p, miR-1292–5p, miR433–3p, miR-10b-3p, and miR-377–5p had significant effects on prognosis (Fig. 1b and S1b). The area under the Receiver Operating Characteristic (ROC) curve (AUC) further indicated that the model with these DEMs had a significant prognostic value (Fig. 1c). The LASSO algorithm was used to further screen for potential prognosis-related DEMs when the minimum mean square error, λ, was 0.04 (Fig. S1a). miR-1292–5p, miR-106a-3p, miR-18b-3p, miR-425–3p, miR-592, and miR-628–5–5p were selected (Fig. 1d and S1c), and the ROC curve indicated that these six miRNAs could influence the prognosis of EEC (Fig. 1e).

Next, nomograms were used for prognostic judgment in above two prognostic models based on the multivariate Cox regression analysis (Fig. 1f, C-index = 0.84, 95 % Confidence Interval [95 %CI 0.77–0.90, *p* < 0.001) and the LASSO Cox regression analysis (Fig. 1 g, C-index = 0.85, 95 % CI 0.80–0.91, *p* < 0.001), showing the favorable prognostic value of both models.

### Identification of independent miRNAs regulated by estradiol in the prognosis of EEC

**Fig. 2:**
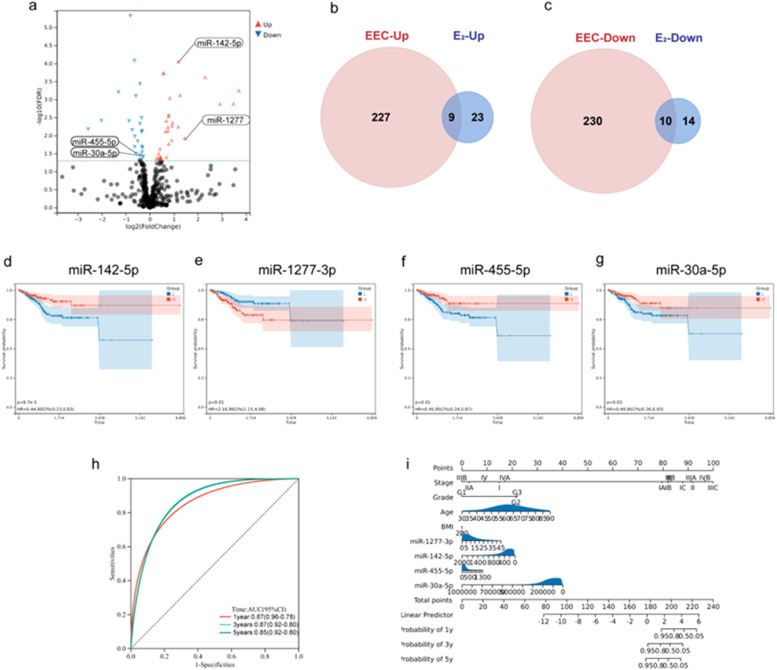
Identification of independent miRNAs for Endometrioid Endometrial Carcinoma (EEC) prognosis regulated by estradiol. (a) Volcano plot of differentially expressed miRNAs (DEMs) in estradiol-treated Ishikawa cells. (b‒c) The Venn diagrams show the overlapping upregulated and downregulated DEMs between EEC tumor tissues and estradiol-treated Ishikawa cells. (d–g) Kaplan–Meier curves for the selected four DEM signatures and the survival time of EEC patients (*p* < 0.05, blue presents expression lower than the median value, red presents expression higher than the median value). (h) The ROC curve of the multivariate Cox regression model with miR-1277–3p, miR142–5p, miR-455–5p, and miR-30a-5p in EEC. (i) The nomogram shows the prognostic model of the above 4 miRNAs.

Considering that estrogen may play an important role in the pathogenesis of EEC, which has been verified in many studies, the authors treated Ishikawa cells with 250 nM estradiol (*n* = 3) or control (*n* = 3), and performed miRNA and mRNA sequencing to explore the effects of estradiol on miRNA regulation and gene expression. Twenty-four downregulated and 32 upregulated miRNAs (|log FC| > 1.0, *P* < 0.05 after FDR adjustment) were identified in estradiol-treated Ishikawa cells (Fig. 2a). Nineteen estradiol-related DEMs were identified by screening the overlapping DEMs between EEC tumor tissues and estradiol-treated Ishikawa cells (Fig. 2b and 2c). Based on these 19 estradiol-related DEMs, the authors used the Kaplan-Meier curve and log-rank test to assess the relationship between miRNA expression and patient survival. Finally, the authors found that elevated miR-1277–3p in EEC predicts poor prognosis, while higher miR-142–5p, miR-455–5p, and miR-30a-5p indicate better outcomes (Fig. 2d‒2 g, *p* < 0.05). Among them, miR-1277–3p and miR142–5p were upregulated, whereas miR-455–5p and miR-30a-5p were downregulated in ECC. Besides, a significant increase in miR-142–5p and a decrease in miR-30a-5p were also observed in the external dataset GSE35794 (Fig. S2a and S2b). Next, the authors constructed a nomogram with these four miRNAs to predict the prognosis of EEC (Fig. 2h‒2i, C-index = 0.84, 95 % CI 0.80–0.89, *p* < 0.001), which revealed the favorable prognosis value (Fig. 2h).

### Function analysis and PPI network construction

**Fig. 3:**
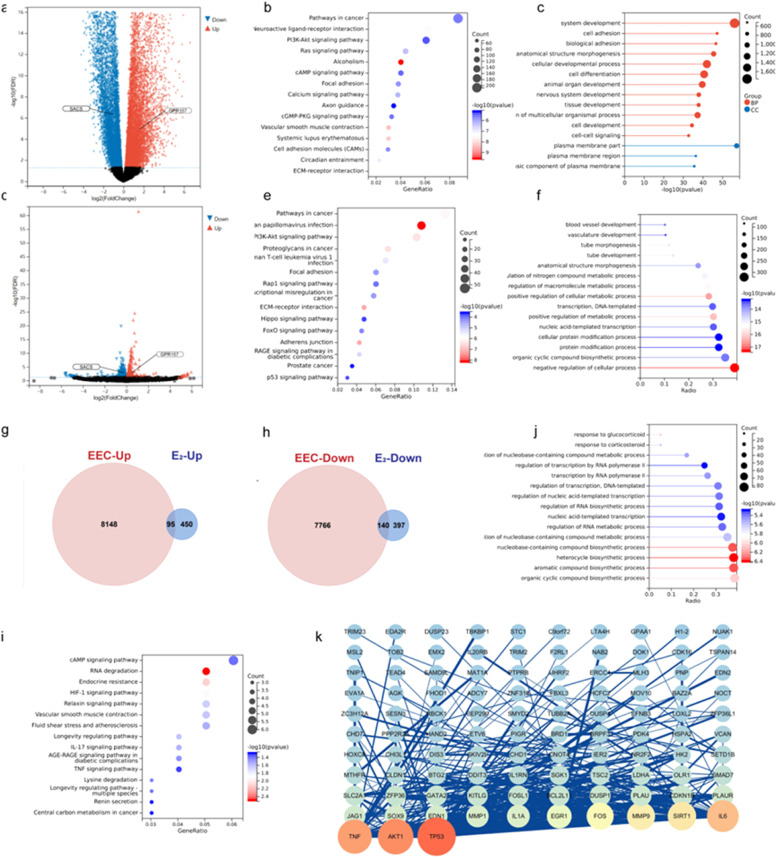
Function analysis and Protein-Protein Interaction (PPI) network construction of Differentially Expressed Genes (DEGs). (a, d) Volcano plot of DEGs in Endometrioid Endometrial Carcinoma (EEC) and in estradiol-treated Ishikawa cells. (g, h) The Venn diagrams show the overlapping upregulated and downregulated DEGs between EEC tumor tissues and estradiol-treated Ishikawa cells. (b, e, i) KEGG pathway analysis of DEGs in EEC, estradiol-treated Ishikawa cells, and the intersection of these two datasets. (c, f, j) GO enrichment analysis of DEGs in EEC, estradiol treated Ishikawa cells, and the intersection of these two datasets. (k) PPI network of the overlapping DEGs between EEC and estradiol treated Ishikawa cells.

To further analyze the role of estradiol in regulating gene expression in EEC, the authors performed differential expression analysis of protein-coding genes based on the TCGA database of EEC (408 tumor tissues and 20 normal tissues) and the mRNA sequencing of Ishikawa cells treated with estradiol or control. The analysis of these gene expression profiles in EEC revealed that 8243 mRNAs were upregulated, and 7906 mRNAs were downregulated (Fig. 3a), whereas 537 downregulated and 545 upregulated genes were identified in estradiol-treated Ishikawa cells (Fig. 3d). The filtering criteria were as described previously (|log FC| > 1.0, *p* < 0.05, after FDR adjustment).

Next, KEGG and GO analyses were respectively performed for the DEGs of ECC and estradiol-treated Ishikawa cells using the “ClusterProfiler” R package. The results showed that the majority of the DEGs were enriched in cancer, PI3k-Akt signaling, and focal adhesion pathways (Fig. 3b and 3e). Furthermore, most of the DEGs in ECC were related to system development and cell adhesion, whereas the DEGs in estradiol-treated Ishikawa cells were mainly enriched in the negative regulation of cellular processes (Fig. 3c and 3f).

Overlapping DEGs (140 downregulated and 95 upregulated genes) between EEC and estradiol-treated Ishikawa cells were chosen for further analysis (Fig. 3 g and 3h). KEGG pathway enrichment analysis indicated that estradiol-regulated DEGs in ECC were mostly enriched in the cAMP signaling pathway, followed by RNA degradation (Fig. 3i). GO analyses showed that most estradiol-regulated DEGs were related to organic cyclic compound biosynthesis (Fig. 3j). Finally, the estradiol regulated DEGs were explored in the PPI network via STRING analysis and 103 genes reached the cutoff criterion (degree > 10) in Cytoscape (Fig. 3k), indicating that TP53, AKT1, and TNF may exert a great impact on the tumorigenesis.

### Establishment of the miRNA-mRNA interaction network and prognostic model

**Fig. 4:**
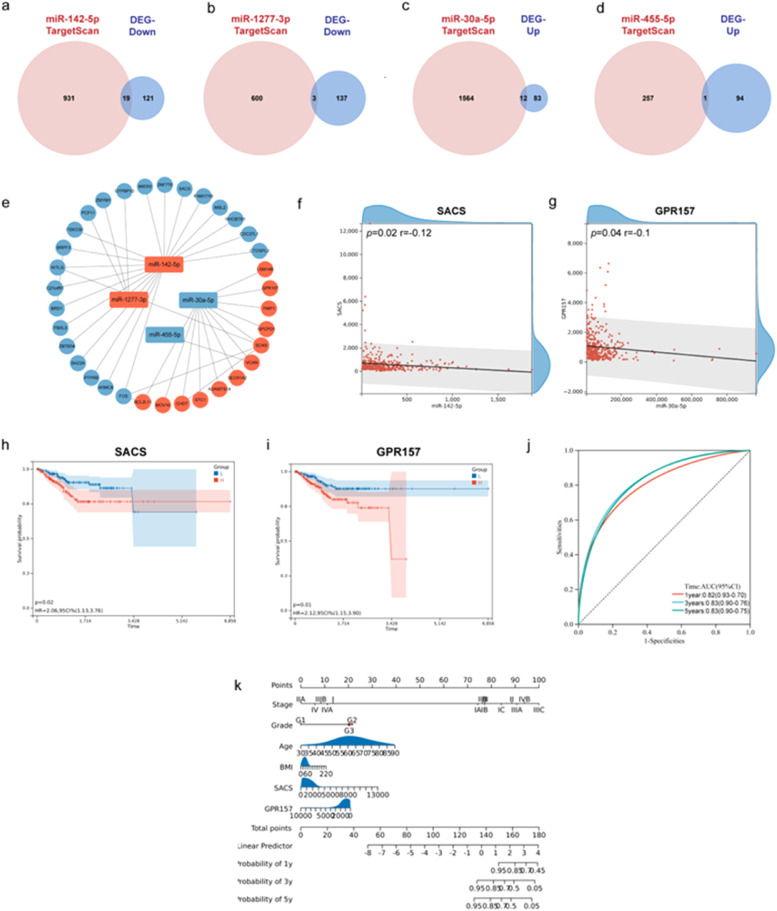
Establishment of the miRNA-mRNA interaction network and the prognostic model for Endometrioid Endometrial Carcinoma (EEC). (a‒d) The Venn diagrams show the overlapping genes between the targets of four selected miRNAs and the intersected DEGs of EEC and estradiol treated Ishikawa cells. (e) Regulatory network map of four selected miRNA and 34 estradiol‐related target genes. (f) Pearson correlation analysis of SACS and miR-142–5p in EEC (*n* = 405). (g) Pearson correlation analysis of GPR157 and miR-30a-5p in EEC (*n* = 405). (h, i) Kaplan–Meier curve for SACS and GPR157 signature and the survival time of patients with EEC (*p* < 0.05, blue presents expression lower than the median value, red presents expression higher than the median value). (j) The ROC curve of the multivariate Cox regression model with SACS and GPR157 in EEC. (k) The nomogram shows the prognostic model with SACS and GPR157.

To explore the possible downstream targets of the estradiol-regulated DEMs, the authors predicted the target genes of miR-142–5p, miR-1277–3p, miR-455–5p, and miR-30a-5p using TargetScan. Next, estradiol-regulated DEGs that were also targeted by these four miRNAs were selected to construct an interaction network (Fig. 4a–4d). These overlapping DEGs were explored in the PPI network using STRING analysis. The miRNA-mRNA network was constructed using Cytoscape software (Fig. 4e). Kaplan-Meier curves and log-rank tests were used to assess the relationship between gene expression and patient survival. Notably, prognostic relevance was demonstrated for SACS (Fig. 4h) and GPR157 (Fig. 4i). Consistent with the mechanisms by which miRNAs regulate mRNA, the correlation analyses showed that miR-142–5p expression was negatively correlated with SACS expression (Fig. 4f), and miR-30a-5p was negatively correlated with GPR157 in EEC (Fig. 4 g).

Therefore, estradiol may influence the prognosis of EEC by upregulating miR-142–5p to reduce SACS, and downregulating miR-30a-5p to increase GPR157. To confirm whether the observed changes in miRNA and mRNA also occurred at other estrogen concentrations, the authors performed miRNA and mRNA sequencing on Ishikawa cells treated with 50 nM estradiol (*n* = 3). Intriguingly, the same alterations in miR-142–5p, miR-30a-5p, SACS, and GPR157 expression were identified, consistent with those observed in the 250 nM estradiol treatment group (Fig. S3a‒S3d). Furthermore, miR-142–5p and miR-30a-5p exhibited consistent expression trends across the added estradiol concentration, suggesting their robust responsiveness to estradiol stimulation. Finally, a nomogram was constructed for prognostic judgment, which showed that this prognostic model with SACS and GPR157 expression was in good agreement with the outcomes of patients with EEC (Fig. 4j and 4k).

### Cell population landscapes and gene expression analysis in EEC and the normal endometrium

**Fig. 5:**
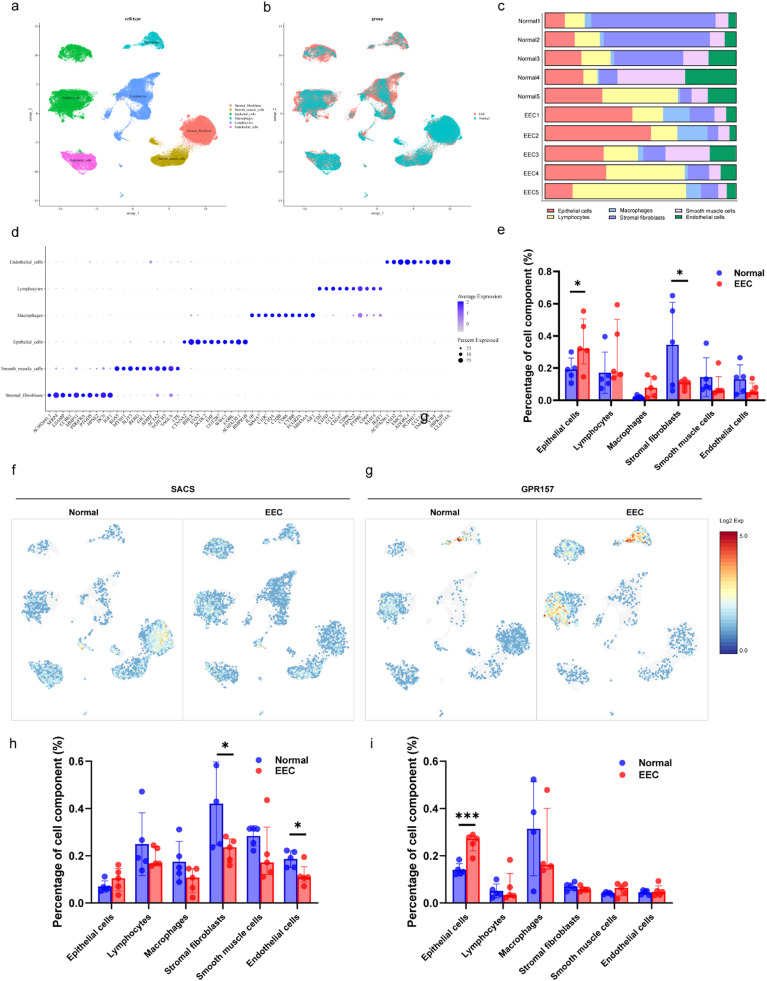
Cell population landscapes of endometrial pathology. (a,b) UMAP plot shows the six main cell type clusters of 10 specimens from normal (*n* = 5) and endometrioid endometrial carcinoma (EEC; *n* = 5) endometrial pathology among the single-cell transcriptome dataset SRP349751. (c) The percentage of six cell types at 10 specimens from normal (*n* = 5) and EEC (*n* = 5) tissue, respectively. (d) Expression patterns of canonical markers and Differentially Expressed Genes (DEGs) of each cell type. Each dot represents a gene, of which the color saturation indicates the average expression level, and the size indicates the percentage of cells expressing the gene. (e) The comparison of the different cell type percentages in normal (*n* = 5) and EEC (*n* = 5) endometrial pathology. Data are mean ± SEM based on five independent biological replicates. Significance was evaluated by comparing it with the normal group (*t*-test, two-sided; p-values are denoted). (f–i) UMAP plot highlighting the expression of gene SACS and GPR157 in normal (*n* = 5) and EEC (*n* = 5) endometrial pathology.

To identify the changes in SACS and GPR157 expression levels in specific cell types, the authors extracted single-cell RNA sequencing data from the SRP349751 dataset, which contained five normal endometrial tissues and five EEC tissues. Thirty clusters were identified (Fig. S4a and S4b) in addition to six known cell types based on previously reported canonical cell markers^17^^,^^18^ (Fig. 5a and 5b): epithelial cells (EPCAM, CDH1) stromal fibroblasts (DCN, COL6A3), endothelial cells (PECAM1, PCDH17), lymphocytes (CCL5, STK17B), macrophages (MS4A6A, CD68), and smooth muscle cells (ACTA2, RGS5). The expression heatmaps of the canonical markers and DEGs for each defined cell type reflected the specificity of the cell population (Fig. 5d). The percentages of epithelial cells and stromal fibroblasts changed drastically between normal endometrial and EEC tissues (Fig. 5c and 5e); the proportion of epithelial cells increased in the EEC, whereas the proportion of stromal fibroblasts dramatically decreased from the normal endometrium to the EEC (Fig. 5e).

Next, SACS and GPR157 are displayed visually in the cell cluster expression diagram (Fig. 5f). Stromal fibroblasts presented the highest SACS expression levels, whereas GPR157 expression levels were higher in epithelial cells and lymphocytes than in other cell types. Interestingly, SACS expression in stromal fibroblasts was significantly reduced, whereas GPR157 expression in epithelial cells was significantly increased in EEC compared to normal tissues. These changes suggested that SACS and GPR157 are involved in EEC development, particularly in the evolution of the cell population.

## Discussion

The incidence of EEC has increased dramatically, threatening women's health worldwide.^1^^,^^19^ It is now the leading cause of cancer-related deaths globally.^3^ As cancer treatment evolves, the authors have entered the era of individualized precision medicine. Next-Generation Sequencing (NGS) technology has accelerated genomic profiling.^20^, ^21^, ^22^ With advancements in science and technology, many oncogenes and tumor suppressor genes have been detected in patients with cancer; however, many unknown genes could be important biomarkers for diagnosis or prognosis.

A growing body of evidence suggests that miRNAs, such as miR-15a-5p^23^ and miR-106a-5p,^24^ serve as superior tumor diagnostic markers compared to other biomarkers due to their higher sensitivity and specificity and that they may play a pivotal role in the prognosis and treatment of EEC.^25^, ^26^, ^27^ More importantly, a single miRNA can directly or indirectly target hundreds of mRNAs, and the miRNA-mRNA network participates in various cellular processes, such as proliferation, apoptosis, and differentiation.^28^, ^29^, ^30^ Therefore, the identification of dysregulated miRNA-mRNAs may aid in understanding the pathogenesis of cancer in addition to its prognostic value. However, few studies have focused on the influence of estrogen on miRNA expression in EEC. Accordingly, in this study, the authors attempted to validate promising diagnostic and prognostic biomarkers via the miRNA–mRNA interaction mechanism, which is regulated by estradiol.

In the present study, the authors identified 19 estradiol-regulated DEMs by comparing endometrial cancer Ishikawa cells treated with 250 nM estradiol or control. Additionally, survival analysis identified two upregulated and two downregulated miRNAs associated with the prognosis of EEC, among which miR-1277–3p was found to be a risk factor; the others (miR142–5p, miR-455–5p, and miR-30a-5p) were protective factors against EEC. Studies have shown that increased expression of miR-142–5p and miR-30a-5p can inhibit Epithelial-Mesenchymal Transition (EMT), reducing tumor metastasis and improving prognosis.^31^, ^32^, ^33^, ^34^ These two miRNAs target key EMT transcription factors, such as ZEB1, SNAI1, and Vimentin, suppressing their expression. miR-142–5p and miR-30a-5p prevent the upregulation of mesenchymal markers (e.g. N-cadherin, Vimentin) and the repression of epithelial markers (e.g. E-cadherin). This inhibition of EMT reduces tumor cell invasion and migration. Furthermore, the authors identified 95 upregulated and 140 downregulated DEGs regulated by estradiol in EEC and screened 103 highly connected target genes from the PPI network, indicating TP53, AKT1, and TNF may play significant roles in the development of ECC regulated by estradiol. Functional enrichment analysis of these genes revealed that they were associated with the cAMP signaling pathway and organic cyclic compound biosynthesis.

To explore the molecular functions of the four prognosis-related miRNAs, the authors predicted their target genes and conducted an in-depth PPI network analysis of the overlapping target genes, including 22 downregulated and 12 upregulated genes. The authors performed the prognostic analysis of 34 overlapping target genes and found that SACS and GPR157 could be used as prognostic biomarkers in patients with EEC. SACS encodes a protein that plays a crucial role in endoplasmic reticulum function and axonal transport.^35^ GPR157, a G-protein coupled receptor, is gaining recognition for its emerging significance in tumor biology.^36^ While its role in EEC remains largely unexplored, early studies suggest it is involved in key cancer-related processes. Notably, SACS is a target gene of miR-142–5p, while GPR157 is regulated by miR-30a-5p Furthermore, in Ishikawa cells treated with 50 nM estradiol, the expression changes of miR-142–5p, miR-30a-5p, SACS and GPR157 were consistent with those observed in the 250 nM estradiol treatment group, suggesting high sensitivity of miR-142–5p and miR-30a-5p to estradiol stimulation.

To identify changes in SACS and GPR157 expression in specific cell types, the authors extracted single-cell RNA-sequencing data from the SRP349751 dataset containing normal endometrial tissues (*n* = 5) and EEC tissues (*n* = 5). The results showed that SACS is mainly expressed in stromal fibroblasts, while GPR157 in epithelial cells and lymphocytes. Meanwhile, the percentage of stromal fibroblasts and the expression level of SACS in stromal fibroblasts were significantly reduced in EEC compared to those in normal tissues, whereas the percentage of epithelial cells and GPR157 expression in epithelial cells increased accordingly. These changes suggest that SACS and GPR157 are involved in the evolution of cell populations during EEC development.

Based on previous studies and analyses of genetic data, the authors presented a comprehensive analysis of the interactions between mRNAs and miRNAs in EEC tissues, constructed a Mrna-miRNA co‐expression network, and finally selected four miRNAs (miR-1277–3p, miR142–5p, miR-455–5p, and miR-30a-5p) and two mRNAs (SACS and GPR157) with prognostic potential in EEC. In addition, functional enrichment analysis of these genes supports their value as biomarkers for disease detection and as an indication of disease progression.

However, the interaction between mRNA-miRNA networks is particularly complex, and bioinformatics‐based analysis is an emerging technology. Further experimental studies are required to validate these findings.

## Conclusion

In conclusion, the authors adopted bioinformatics methods to systematically analyze EEC‐related mRNAs and miRNAs in the TCGA database (*n* = 408) and the original sequencing, which performed on Ishikawa cells treated with 250 nM estradiol (*n* = 3), 50 nM estradiol (*n* = 3) or control (*n* = 3). Four miRNAs and two target genes regulated by estradiol were identified as potential biomarkers for the prognosis of patients with EEC. These findings improve the understanding of the pathogenesis and potential molecular events of EEC, contribute to the timely diagnosis and prognosis of patients with EEC, and lay a foundation for future clinical research.

## Authors’ contributions

All authors participated in the design, interpretation of the studies, analysis of the data, and review of the manuscript. LJ and QX jointly contributed to the conceptualization of the research framework, funding acquisition to support the project, and supervision throughout its implementation. QX additionally led the methodology design and performed formal analysis of the data. JTH played a key role in methodology development, experimental execution, and formal analysis, while taking primary responsibility for drafting the initial manuscript. YX and JH contributed to formal analysis, supporting data interpretation. All authors read and approved the final manuscript.

## Fundings

This study was supported by grants from the 10.13039/501100001809National Natural Science Foundation of China (81871146), the 10.13039/501100012232National Key Clinical Specialty Construction Project (U114000) and the National High-Level Hospital Clinical Research Funding (2022-PUMCH-A-206).

## Declaration of competing interest

The authors declare no conflicts of interest.

## Appendix Supplementary materials

Image, application 1
