# Supplementary material for: Identification of prognostic biomarkers for endometrioid endometrial carcinoma based on the miRNA and mRNA co‐expression network regulated by estradiol
Source: Clinics (Sao Paulo). 2025 May 3;80:100672. doi: 10.1016/j.clinsp.2025.100672 (PMC12098172; doi:10.1016/j.clinsp.2025.100672)
Supplement: Supplementary file 1 [file mmc1.docx]

**CLINICS-D-24-00833_ Supplementary Material**

**Figure** **Supplementary S1 Prognostic models based on the multivariate Cox regression and the LASSO Cox regression.** (a) The optimal λ-values are indicated by the vertical black lines, and a λ-value of 0.04 was selected. (b, c) The heatmap of the expression level of the chosen miRNAs in EEC patients alive or dead.

**Figure Supplementary S2 The expression of miR-142-5p and miR-30a-5p in dataset GSE35794.** (a‒b) Log2 expression of miR-142-5p and miR-30a-5p in dataset GSE35794, comprising 11 EEC samples and 3 normal controls.

**Figure Supplementary S3 The expression of miRNA and mRNA in groups treated with different concentrations of estradiol.** (a‒b) Expression of miR-142-5p or miR-30a-5p in Ishikawa cells treated with 250 nM estradiol (n = 3), 50 nM estradiol (n = 3) or control (n = 3). (c‒d) Expression of SACS or GPR157 in Ishikawa cells treated with 250 nM estradiol (n = 3), 50 nM estradiol (n = 3) or control (n = 3).

**Figure Supplementary S4 UMAP plot showing cell clusters.** (a) Clustering of high-quality cells from normal (n = 5) and endometrioid endometrial carcinoma (EEC; n = 5) endometrial pathology among the single-cell transcriptome dataset SRP349751. (b) UMAP plot of six main cell type clusters from every normal (n = 5) and EEC (n = 5) sample.
